# Bacterial virulence traits: A potential area of study for drug development

**DOI:** 10.4103/0975-7406.72145

**Published:** 2010

**Authors:** Roopesh Jain, Susmit Kosta, Archana Tiwari

**Affiliations:** School of Biotechnology, Rajiv Gandhi Proudyogiki Vishwavidyalaya, Airport Bypass Road, Gandhi Nagar, Bhopal-462 036, Madhya Pradesh, India. E-mail: skosta@webdunia.com

Sir,

The study of virulence traits among the bacteria represents an upcoming focus in the field of bacterial pathogenesis, where another serious emerging health concern today is the appearance and spread of resistant bacterial stains.[1] It has been reported by several scientists during the last two decades that bacteria exhibit many of the similarities in terms of mechanism and adaptation with multicellular organisms, when they come in contact with them. This behavior provides multiple expressions of virulence that individual free-swimming bacteria do not possess.[2] Basically, the two types of virulence traits in terms of communal and individual microbes are: (i) chronic or persistent infections, which are largely associated with populations of microbes and (ii) individual bacterial virulence traits, associated with acute infections. In acutal sense, the study of “Virulence Mechanisms of Pathogens” is a discipline that emphasizes on the mechanisms of host–pathogen interactions, focusing on the mechanisms used by pathogens to establish infection, produce disease and persist in the host.[3] Molecular aspects of virulence in pathogens start with the basic study of bacterial invasion, colonization and survival, which includes pathogen adherence, invasion on different line of body defense, evasion of host-derived antimicrobial peptides, and the contribution of antibiotic resistance to bacterial survival in the host and regulation of virulence genes.[4] The strategies that the pathogens use to invade host defense mechanisms pursuing their survival within macrophages, bacterial resistance to antibodies, effect on nucleic acid and virulence are interesting aspects of the study.[5]

Current knowledge on pathogen’s effects on host cell function, induction of secretory pathway that many pathogenic bacteria use to export virulence-related proteins, regulation of virulence genes and identification of these by analysis of bacterial DNA sequence data are the proof of revolution in modern science and advanced technology.[6] It has given immeasurable rhythm to this area of research and has led to an avalanche of new information. Present scenario of pathogenesis study indicates that during disease process, pathogens are heavily exposed to different specific and non-specific host defense mechanisms and other adverse conditions related to their survival, for instance, stress and drug effects. To exist under these adverse conditions, pathogens use well-directed strategies controlling their expression of virulence factors in response to host-induced environmental changes and immune defense. Small regulatory mechanisms involving many regulatory molecules like small RNA, sigma factors, etc. play an important role in pathogenesis. Despite progress in our understanding of all these disease biology facts, information related to microbial virulence remains enigmatic. Even little is known about diversity and abundance of all type of pathogens within the human body or the types of their interactions with human cells and components of the human immune system, or other members of the endogenous flora. These are few hot topics that attract the scientists nowadays.

Another important issue that needs serious attention is the understanding of evolution of pathogens, considering their phylogenetic distribution, while the genetic and evolutionary factors that have contributed to their emergence are important to find out spatial virulence. The comparison of spatial virulence worldwide will definitely solve related mechanism of virulence to discover the strategies used by bacteria to subvert immune defences and cause disease.[7] A detailed study will definitely identify universal targets for global drug development to treat and prevent infectious diseases. The value of this kind of research for developing countries like India, Pakistan, Bangladesh and developing countries of African continent, where disease rate is too high, will be immense to provide cheap drugs and new multimodality therapies.

## References

[1] Chapin A, Rule A, Gibson K, Buckley T, Schwab K (2005). Airborne multidrug-resistant bacteria isolated from a concentrated swine feeding operation. Environ Health Perspect.

[2] Hu FZ, Ehrlich GD (2008). Population-level virulence factors amongst pathogenic bacteria: Relation to infection outcome. Future Microbiol.

[3] Konkel ME, Tilly K (2000). Temperature-regulated expression of bacterial virulence genes. Microbes Infect.

[4] Shin S, Roy CR (2008). Host cell processes that influence the intracellular survival of Legionella pneumophila. Cell Microbiol.

[5] Wu HJ, Wang AH, Jennings MP (2008). Discovery of virulence factors of pathogenic bacteria. Curr Opin Chem Biol.

[6] Lambris JD, Ricklin D, Geisbrecht BV (2008). Complement evasion by human pathogens. Nat Rev Microbiol.

[7] Briken V (2008). Molecular mechanisms of host-pathogen interactions and their potential for the discovery of new drug targets. Curr Drug Targets.

